# Cytokine concentration in peripheral blood of patients with childhood obesity

**DOI:** 10.3389/fimmu.2025.1606375

**Published:** 2025-05-30

**Authors:** Jia Mai, Min Wang, Ying Guo, Ling Yang, HongJian Xie, Lan Mei, ZiYao Zhu, XiaoJuan Liu

**Affiliations:** Department of Laboratory Medicine, West China Second University Hospital, Sichuan University; Key Laboratory of Birth Defects and Related Diseases of Women and Children (Sichuan University), Ministry of Education, Chengdu, Sichuan, China

**Keywords:** cytokine concentration, peripheral blood, childhood obesity, inflammation, IL-6

## Abstract

**Introduction:**

Obesity is marked by chronic inflammation, with research showing notable changes in cytokines. However, systematic investigations into cytokine level changes are still lacking. This study compared the concentrations of various cytokines in the peripheral blood of healthy controls and obese children.

**Methods:**

Peripheral blood samples from test cohort including 5 healthy children and 5 obesity children were used to evaluate concentration levels of 48 inflammatory cytokines by Bio-Plex assay. Diet-induced obese (DIO) mice used to assess whether there were significant differences in the expression of MIP-1b, PDGF-BB. Peripheral blood samples from validation cohort including 44 healthy children and 44 obesity children were used to evaluate concentration levels of MIP-1b, PDGF-BB, IP-10, IL-6, IL-9 and TNF-β.

**Results:**

In the Bio-Plex assay, there were significant differences in MIP-1b and PDGF-BB levels between healthy and obesity children. Additionally, IP-10, IL-6, IL-9, and TNF-β exhibited a trend of difference between the two groups. However, no significant differences in MIP-1b and PDGF-BB levels were observed between control and DIO mice. Notably, we found that IL-6 exhibited a significant concentration difference between the serum of healthy and obesity children, suggesting the IL-6 may play a key role in the inflammatory response associated with obesity.

**Discussion:**

In summary, our study emphasizes the importance of IL-6 and other cytokines in childhood obesity research and suggests that future investigations should further explore the specific roles of these cytokines in the pathophysiological states associated with obesity, aiming to provide new strategies for the prevention and treatment of obesity.

## 1 Introduction

The global prevalence of obesity has witnessed a significant upsurge, with an estimated 600 million adults and 100 million children meeting the criteria for obesity. This issue is widely acknowledged as a substantial public health challenge (1). Obesity is a chronic condition resulting from the complex interplay of genetic and environmental factors, leading to dysregulation of energy metabolism and subsequent excessive adipose tissue accumulation or aberrant fat distribution (2, 3). In recent decades, there has been a marked escalation in the prevalence of obesity, evident not only among adolescents but also among children (4). It is widely acknowledged that obesity and its comorbidities such as hypertension, dyslipidemia, diabetes, and cardiovascular disease tend to endure into adulthood (5), so the prevention for children obesity is essential and significant.

Obesity is intricately associated with chronic low-grade inflammation in adipose tissue, especially white adipose tissue (WAT). This sustained inflammatory response, triggered by metabolic signals, can impede insulin function, disrupt metabolic equilibrium, and represents a pivotal link between obesity and related conditions such as insulin resistance and type 2 diabetes (6). The imbalance of inflammatory factors contributes to the pathogenesis of obesity and related diseases by inducing chronic inflammation in adipose tissue. Cytokines are small protein molecules synthesized and secreted by immune cells (including T cells, B cells, monocytes, macrophages, and natural killer (NK) cells) and some non-immune cells (such as epithelial cells, endothelial cells, and fibroblasts) in response to stimulation. Cytokines attach to particular receptors and modulate immune responses to govern cell growth, differentiation, and function. In the context of obesity, it was initially believed that adipocytes were the primary source of obesity-related cytokines (7). However, with the discovery of macrophages in adipose tissues, it has become evident that macrophages and various other immune cells are the main inflammatory cell types responsible for releasing most of the inflammatory molecules in both animals and humans with obesity (8–10).

The initial indication of cytokine associated with obesity was found in a study that reported heightened levels of tumor necrosis factor-alpha (TNF-α) in adipose tissue in individuals with obesity (11). Subsequent research has consistently demonstrated an escalation of inflammation in adipose tissue among both animals and humans with obesity (8). Previous studies have shown that there is a significant increase in the expression level of interleukin-6 (IL-6), which contributes to heightened insulin resistance and an elevated risk of cardiovascular complications. The secretion of other cytokines, such as interleukin 1 beta (IL-1b), interleukin 18 (IL-18), C-C motif chemokine ligand 2 (CCL2, MCP-1) and interleukin 8 (IL-8) are also contributing factors to the exacerbation of local inflammation and the inhibition of insulin sensitivity (12, 13).

Most research on circulating inflammatory indicators has concentrated on a narrow set of possible markers. However, these indicators only make up a part of the whole cascade of inflammation. Inflammatory processes are intricate reactions to external stimuli that entail the interplay of signaling molecules, including growth factors, angiogenic factors, pro- and anti-inflammatory cytokines, and chemokines, and host cells. There haven’t been many thorough investigations on the connection between these circulating cytokines and the onset and advancement of childhood obesity. Hence, in this study, we carried out a case-control investigation to compare the levels of 48 serum cytokines in patients with childhood obesity with those health children.

## 2 Methods

### 2.1 Patients and specimens

Between 2023 and 2024, peripheral blood samples were collected from 49 health children and 49 children diagnosed with obesity. The recruitment process and clinical characteristics of the participants are detailed below. Health children were primarily identified through routine childhood checkups conducted at West China Second University Hospital. These checkups included comprehensive physical examinations and medical history reviews. Children with obesity were identified through routine checkups and referrals from pediatricians and nutritionists. Many of these children were referred for further evaluation and management of obesity-related complications. Inclusion criteria were: children aged 0–18 years with BMI ≥ 95th percentile for age and sex were included as obesity group. Healthy controls were children aged 0–18 years with a BMI between the 5th and 85th percentiles. Parents of these children were evaluated for tobacco smoking status, and none of the parents were active smokers. None of the children had a history of asthma or other chronic inflammatory conditions. Exclusion criteria were children with acute infections, autoimmune diseases, or other chronic illnesses (e.g., diabetes, thyroid disorders). Informed consent was obtained from the legal guardians of all participants. The study protocol, including the consent process was approved by the Ethics Committee of West China Second University Hospital (approval number: 2022100). The sample size was calculated using GPower based on preliminary data for IL-6. For a two-tailed t-test with α =0.05 and power (1-β) = 0.80, the required sample size was determined.

### 2.2 Bio-plex multiplex cytokine assay

Serum cytokine levels were quantified using the Bio-Plex Pro Human Cytokine Screening 48-plex Panel kit (#12007283, Bio-Rad Laboratories, California, USA), which includes 48 analytes (**Supplementary Table S1**). The assays dynamic ranges, sensitivities (LOD: 0.05 to 141.77 pg/mL), analytes below the lower limit were excluded. The inter-/intra-assay CVs (<10%) are detailed in **Supplementary Table S1**. Sample were diluted 1:4, and data acquired on a Bio-Plex 200 system. The assay was conducted according to the manufacturer’s protocol (Wayen Biotechnologies, Shanghai, China). The 48 analytes including: Basic FGF, CTACK, Eotaxin, G-CSF, GM-CSF, GRO-a, HGF, IFN-a2, IFN-g, IL-1a, IL-1b, IL-1ra, IL-2, IL-2Ra, IL-3, IL-4, IL-5, IL-6, IL-7, IL-8, IL-9, IL-10, IL-12 (p40), IL-12 (p70), IL-13, IL-15, IL-16, IL-17A, IL-18, IP-10, LIF, MCP-1 (MCAF), MCP-3, M-CSF, MIF, MIG, MIP-1a, MIP-1b, b-NGF, PDGF-BB, RANTES, SCF, SCGF-b, SDF-1a, TNF-α, TNF-β, TRAIL and VEGF-A.

### 2.3 Mice

Animal experiments were performed in accordance with animal care ethics approval and guidelines of the Ethics Committee of West China Second University Hospital (approval number: 202307045). C57BL/6J male mice (6-week-old) were purchased from Beijing Vital River Laboratory Animal Technology Co., Ltd and randomly assigned to two groups that feed a standard diet or a high-fat-diet (60% kcal fat, D12492) from 8 weeks of age. All animals were housed under controlled environmental conditions (20-26°C, 12h/12h light-dark cycle). Sample size was determined based on prior studies (n=5-8/group) to achieve 80% power (α=0.05). Every 6 mice were in one group, and every 3 mice were in one cage. The body weight of mice was measured weekly. Diet-induced obesity develops after ≥ 10 weeks of high fat diet and obesity was defined as ≥ 20% increased body weight relative to wild-type controls. At the age of 19 weeks of mice, the blood and fat specimens of obesity group and the control group of mice were collected. After the mice were anesthetized, the orbital blood was collected. Try to collect as much blood as possible and avoid hemolysis. After the blood specimens were collected, the serum was separated by centrifugation and used for the detection of HDL, LDL, TC, MIP-1b, PDGF-BB. The mice were sacrificed by cervical dislocation, and subcutaneous fat and epididymal fat (visceral fat) of the mice were surgically collected.

### 2.4 Enzyme-linked immunosorbent assay

Mice serum MIP-1b, PDGF-BB and human validation set serum MIP-1b, PDGF-BB, IP-10, IL-6, IL-9 and TNF-β were quantified using ELISA kits (Elabscience Biotechnology Co,. Ltd). The assay was conducted according to the manufacturer’s protocol. Briefly, 96-well plates pre-coated with capture antibodies were incubated with standards/serum (100μL/well). After washing, biotinylated detection antibodies (100μL/well) and HRP (100μL/well) were added. Colorimetric detection used substrate (15min) followed by stop solution (50μL). ELISA detection limits: mice serum MIP-1b (9.38 pg/mL), PDGF-BB (23.44 pg/mL) and human serum MIP-1b (18.75 pg/mL), PDGF-BB (18.75 pg/mL), IP-10 (4.69 pg/mL), IL-6 (0.49 pg/mL), IL-9 (9.38 pg/mL) and TNF-β (9.38 pg/mL).

### 2.5 Laboratory measurements of peripheral blood lipid metabolism indices

HDL, LDL, ApoA, ApoB and TC were assessed with Siemens ADVIA Chemisty XPT according to the vendors’ protocols.

### 2.6 Statistical analysis

Quantitative data were compared using two-tailed unpaired Student’s t-test. Associations between cytokines were assessed using Pearson’s chi-square test. Receiver operating characteristics (ROC) curves were constructed to assess sensitivity, specificity, and respective areas under the curves (AUCs) with 95% CI. We investigated the optimum cutoff value for diagnosis by maximizing the sum of sensitivity and specificity. Statistical significance was set at *p*<0.05. All data were analyzed using GraphPad Prism 8.0, R studio 4.3.3, SPSS 20.0 and Microsoft Excel.

## 3 Results

### 3.1 Cytokine concentrations in serum in the test cohorts

**Figure 1:**
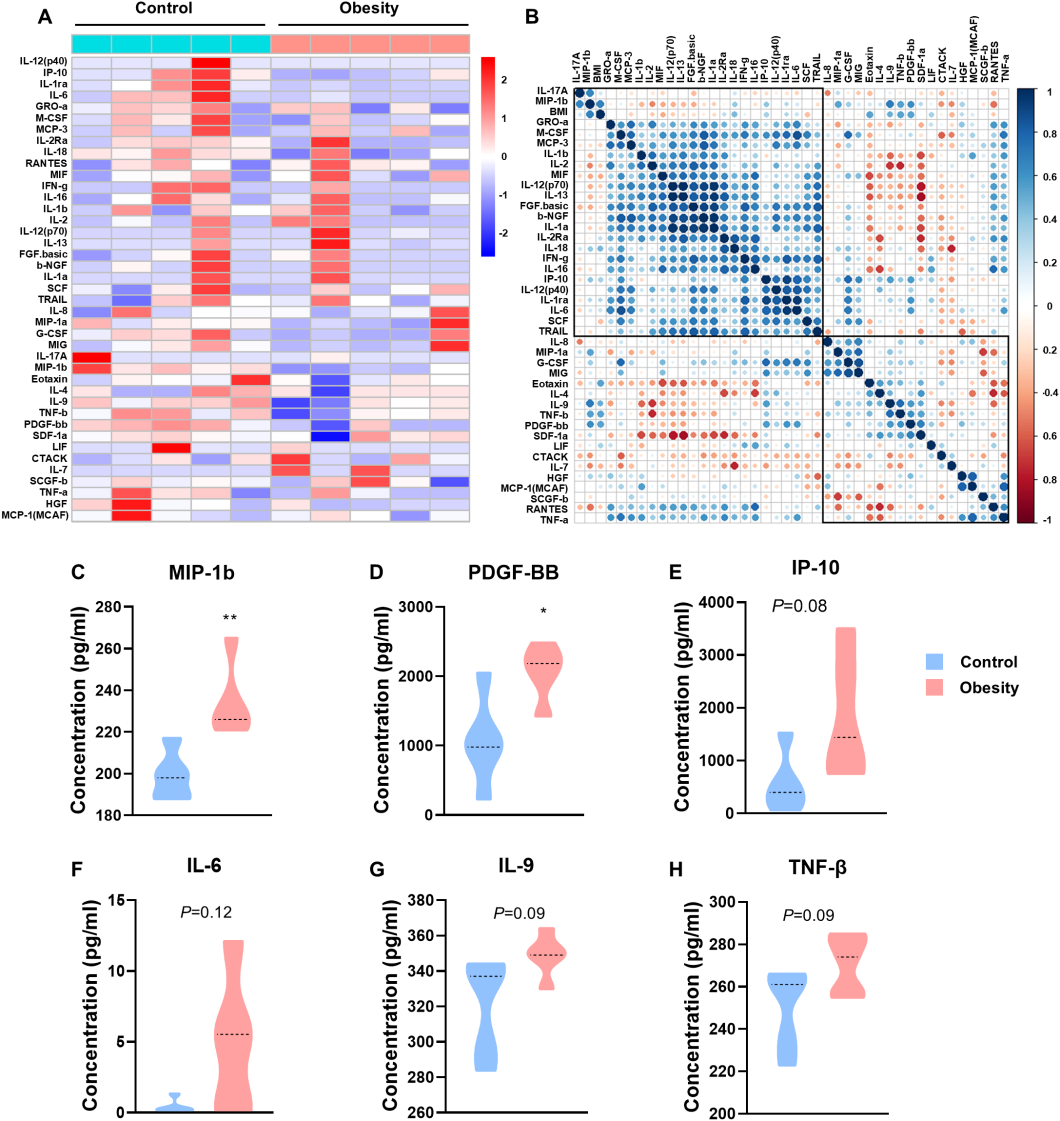
The concentrations of cytokines in patients with childhood obesity. **(A)** Heatmap of differentially expressed 41 serum multi-cytokines between participants with childhood obesity and healthy controls using Bio-Plex assay. **(B)** Correlation heatmap of 41 serum cytokines and BMI in healthy controls and children with obesity. **(C-H)** Concentrations of MIP-1b **(C)**, PDGF-BB **(D)**, IP-10 **(E)**, IL-6 **(F)**, IL-9 **(G)** and TNF-β **(H)** in the peripheral blood of the test cohort. (*: *P*<0.05; **: *P*<0.01).

We recruited 98 participants overall, 10 in the test cohort and 88 in the validation cohort. We employed a Bio-Plex multiplex cytokine assay to determine the concentration of serum cytokines in the patients of the test cohort and identified the cytokines with significant differences. The ELISA method was utilized for verification in the serum of the patients in the validation cohort. The clinicopathological characteristics of the patients are summarized in **Table 1**. According to the vendor-recommended program, 48 candidate serum cytokines were selected for evaluation. As result, concentration of 7 cytokines including: GM-CSF, IFN-a2, IL-3, IL-5, IL-10, IL-15 and VEGF were less than the detection range. The heat map shows the concentrations of other 41 cytokines in the 10 test cohort serum samples (**Figure 1A**). The correlation heat map showed the correlation between the 41 cytokines (**Figure 1B**).

**Table 1:** The clinicopathological characteristics of the patients with childhood obesity and healthy controls.

Characteristics	Control (n=49)	Obesity (n=49)	P value
	Mean±SD	Mean±SD	
Sex (F/M)	28/21	35/14	NS
Age (years)	9.08±3.46	10.43±2.74	NS
BMI (kg/m²)	15.86±3.48	25.51±4.65	<0.001
TC (mmol/L)	4.37±1.49	4.05±0.89	NS
TG (mmol/L)	0.83±0.44	1.18±0.56	<0.001
HDL (mmol/L)	1.53±0.43	1.25±0.36	<0.001
LDL (mmol/L)	2.72±1.30	2.57±0.98	NS
ApoA (g/L)	1.39±0.29	1.29±0.25	NS
ApoB (g/L)	0.69±0.26	0.68±0.22	NS

The concentrations of C-C motif chemokine ligand 4 (CCL4/MIP-1b) and platelet derived growth factor BB (PDGF-BB) were significantly higher in patients with childhood obesity than in control group (MIP-1b, *p*=0.0082, **Figure 1C**; PDGF-BB, *p*=0.0228, **Figure 1D**). Additionally, the concentrations of interferon gamma induced protein 10kDa (CXCL10/IP-10), interleukin 6 (IL-6), interleukin 9 (IL-9) and tumor necrosis factor beta (TNF-β) also showed a tendency higher in patients with childhood obesity than in control group (IP-10, *p*=0.0823, **Figure 1E**; IL-6, *p*=0.1163, **Figure 1F**; IL-9, *p*=0.0922, **Figure 1G**; TNF-β, *p*=0.0879, **Figure 1H**). These cytokines failed to exhibit significantly differences potentially due to the overly small sample size. We will employ a larger sample size in the validation set for verification.

### 3.2 MIP-1b and PDGF-BB showed no significant difference in the serum of normal and obesity mice

**Figure 2:**
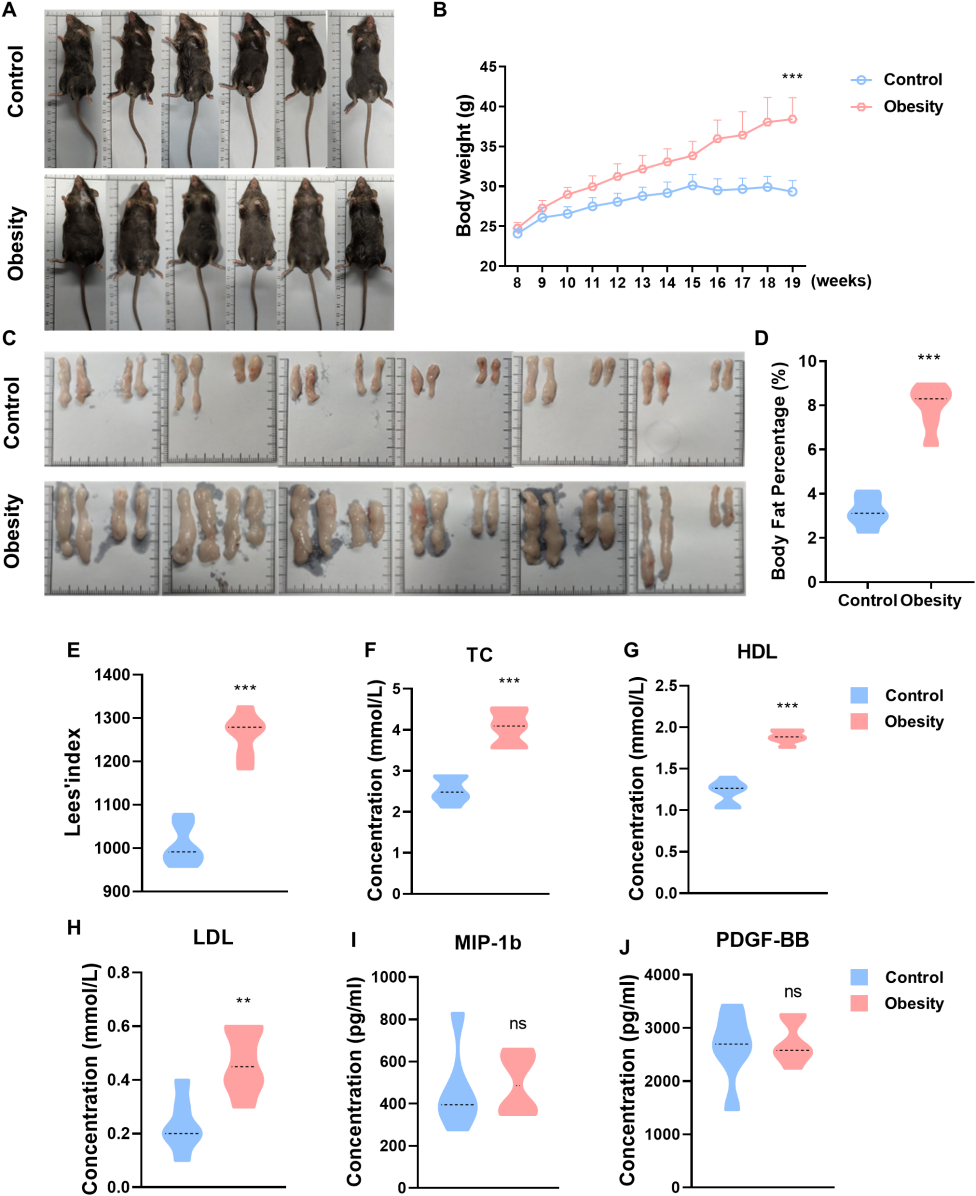
The concentrations of cytokines in the serum of control and obesity mice. **(A)** Representative photo in C57BL/6J mice fed standard diet (control) or high-fat-diet (obesity) for 19 weeks. **(B)** Growth curve in body weight of standard diet (control) or high-fat-diet fed (obesity) C57BL/6J mice. **(C)** Subcutaneous and epididymal adipose tissue of standard diet or high-fat-diet fed C57BL/6J mice. **(D)** Body fat percentage of control and obesity mice. **(E)** Lees’ index of control and obesity mice. **(F-H)** Blood lipid metabolism markers levels of control and obesity mice. **(I, J)** Concentrations of MIP-1b **(I)**, PDGF-BB **(J)** in the serum of control and obesity mice. (ns: *P*>0.05, not significant; **: *P*<0.01; ***: *P*<0.001).

In order to multifaceted learning the variation of the most obviously changing cytokine, we established the model of mouse with obesity by long-term feeding with high fat diet. The two groups of mice were fed with standard diet (control group) and high fat diet (obesity group), from 8 to 19 weeks of age for 11 weeks (**Figure 2A**). Continuous long time (11weeks), obesity group resulted in obvious higher body weight in mice as compared with control group mice (**Figure 2B**). Subcutaneous and epididymal adipose tissue were extracted through surgical intervention for weighing, and the body fat percentage was computed based on body weight and total adipose tissue mass (**Figure 2C**). The experimental outcomes indicated that the body fat percentage of mice with obesity induced by high fat diet was conspicuously higher than that of normal mice (**Figure 2D**). Lees’ index is an indicator for measuring the degree of obesity in mice, and the calculation formula thereof is body weight (g)/body length (cm)×1000. As expect, the Lees’ index of obesity group was significantly higher than that of control group mice (**Figure 2E**). Blood lipid tests showed that the serum total cholesterol (TC), high-density lipoprotein (HDL) and low-density lipoprotein (LDL) levels of mice in obesity group were significantly higher than that of control group (**Figures 2F-H**). The above results evince that the modeling of mice with obesity in our study was successful. And then, ELISA assay was used to test the most obviously changing cytokine of Bio-Plex assay: MIP-1b and PDGF-BB. However, it is mostly likely due to the small sample size that there is no significant difference in the concentration of MIP-1b and PDGF-BB in the serum of normal and obesity mice (**Figures 2I, J**).

### 3.3 Cytokine concentrations in serum in the validation cohorts

**Figure 3:**
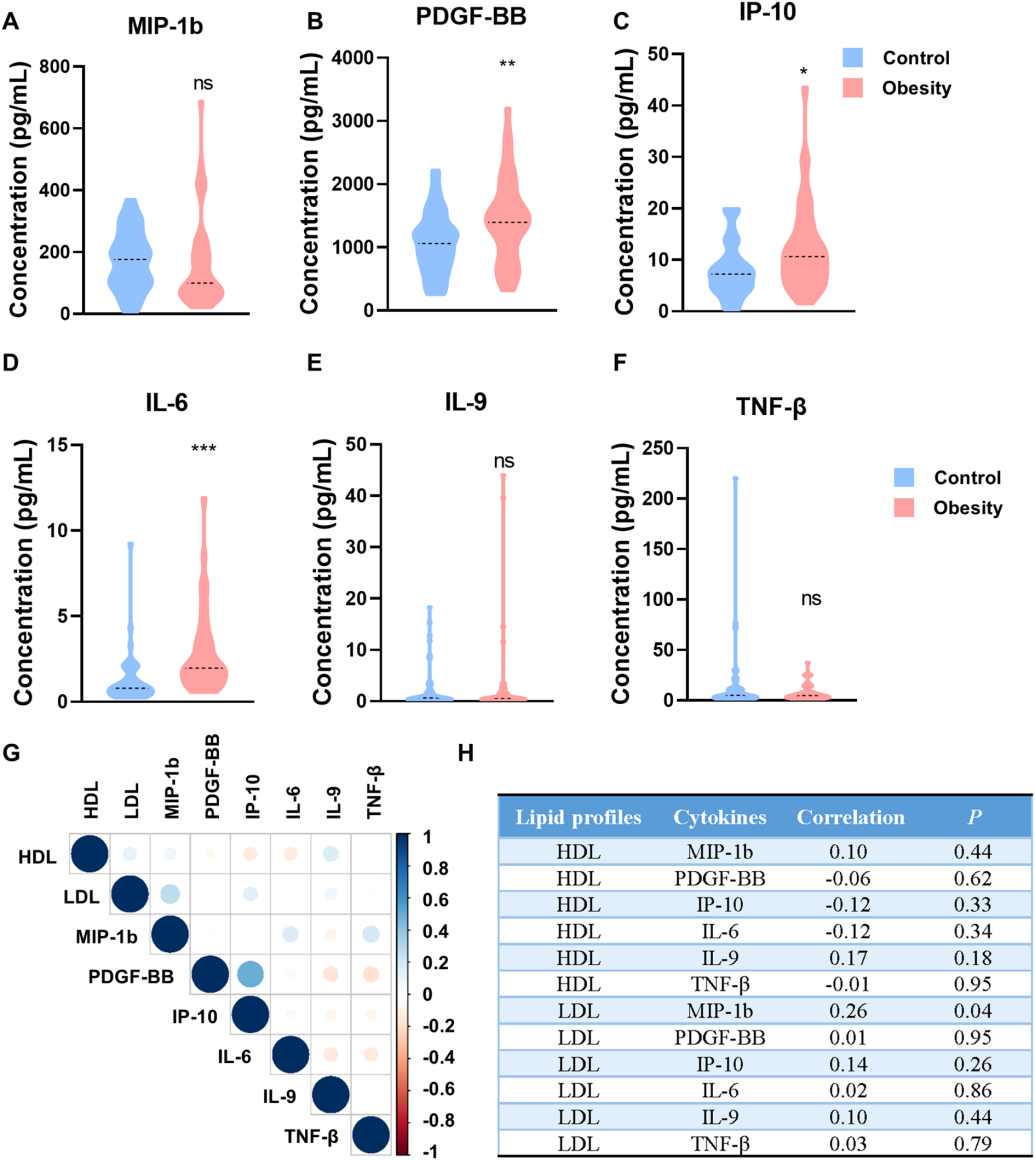
The concentrations of cytokines in patients with childhood obesity of validation cohorts. **(A-F)** Concentrations of MIP-1b **(A)**, PDGF-BB **(B)**, IP-10 **(C)**, IL-6 **(D)**, IL-9 **(E)** and TNF-β **(F)** in the peripheral blood of the validation cohorts. **(G)** Correlation heatmap of HDL/LDL and individual cytokines (MIP-1b, PDGF-BB, IP-10, IL-6, IL-9 and TNF-β). **(H)** The correlation index and *P* value of HDL/LDL and individual cytokines (MIP-1b, PDGF-BB, IP-10, IL-6, IL-9 and TNF-β). (ns: *P*>0.05, not significant; *: *P*<0.05; **: *P*<0.01; ***: *P*<0.001).

A total of 88 participants were included in validation cohorts, comprising 44 health children and 44 obesity children. ELISA assay was used to determine the concentration of cytokines in the serum, including MIP-1b, PDGF-BB, IP-10, IL-6, IL-9 and TNF-β. The concentrations of MIP-1b, IL-9 and TNF-β shown no significant difference in the serum of health and obesity children (MIP-1b, **Figure 3A**; IL-9, **Figure 3E**; TNF-β, **Figure 3F**). Although numerous research had reported an increase in MIP-1b, IL-9 and TNF-β in obesity, our experimental findings suggest that the elevation of MIP-1b, IL-9 and TNF-β are not pronounced in the serum of obesity children. Additionally, the concentrations of PDGF-BB, IP-10 and IL-6 were significantly higher in patients with childhood obesity than in control group (PDGF-BB, *p*=0.0045, **Figure 3B**; IP-10, *p*=0.0393, **Figure 3C**; IL-6, *p*<0.0001, **Figure 3D**). Among all the detected cytokines, the alteration of IL-6 is the most conspicuous, indicating that IL-6 can serve as one of the diagnostic indicators for childhood obesity. Even more significantly, IL-6 shows great potential as an early warning indicator for childhood obesity.

We further analyzed the correlation between cytokine levels and lipid profiles, including HDL and LDL. Although no significant direct correlations were observed between individual cytokines (MIP-1b, PDGF-BB, IP-10, IL-6, IL-9 and TNF-β) and HDL/LDL levels in this study (**Figures 3G, H**), the overall trend of increased pro-inflammatory cytokines in obese children suggests a potential link between chronic inflammation and metabolism dysregulation.

### 3.4 Diagnostic efficiency of PDGF-BB, IP-10 and IL-6 in childhood obesity

BMI serves as the most straightforward indicator for diagnosing obesity and it is the principal basis for diagnosing childhood obesity in clinical practice. Once obesity is instantiated, intervention and treatment are extremely challenging. To tackle the problem of childhood obesity, the core lies in early prevention (14). Thus, looking for early warning indicators of childhood obesity is of great significance for the prevention of childhood obesity. In this study, although the serum specimens of the children we selected were from those who had already developed obesity, they still could exert a certain indicative effect on the alterations of the early indicators of obesity. Hence, we calculated the diagnostic efficacy of these indicators to provide a certain foundation for their role as early indicators.

**Figure 4:**
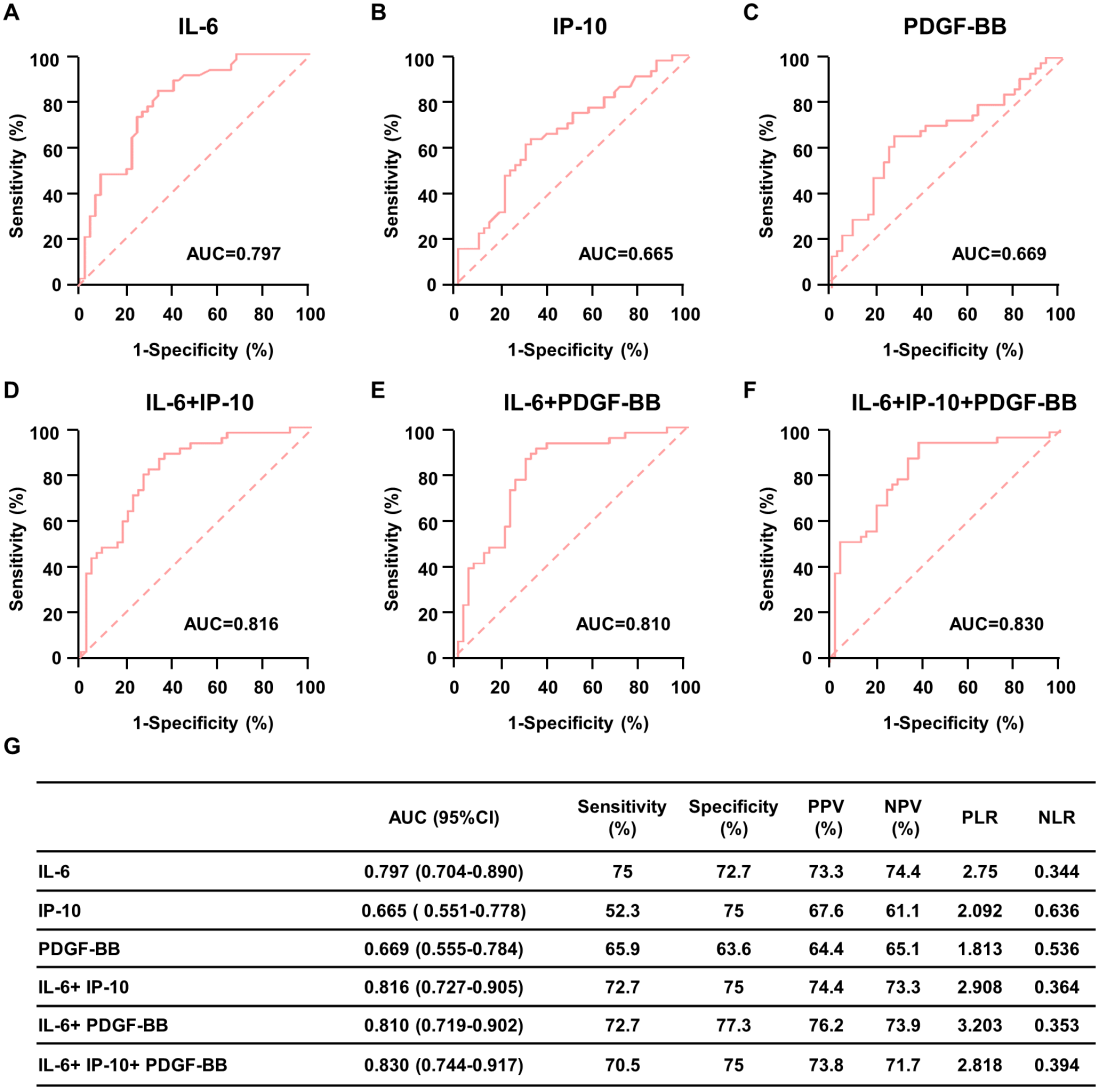
Diagnostic outcomes for serum cytokines in the diagnosis of childhood obesity. **(A-C)** ROC curve for IL-6 **(A)**, IP-10 **(B)** and PDGF-BB **(C)** for patients with childhood obesity versus controls in the validation cohorts. **(D)** ROC curve for both IL-6 and IP-10 for patients with childhood obesity versus controls in the validation cohorts. **(E)** ROC curve for both IL-6 and PDGF-BB for patients with childhood obesity versus controls in the validation cohorts. **(F)** ROC curve for combine IL-6, IP-10 and PDGF-BB for patients with childhood obesity versus controls in the validation cohorts. **(G)** Results for measurement of serum IL-6, IP-10, PDGF-BB, or both in the diagnosis of childhood obesity, AUC (area under curve); PPV (positive predictive value); NPV (negative predictive value); PLR (positive likelihood ratio); NLR (negative likelihood ratio).

ROC curves showed the optimum diagnostic cutoff for IL-6 was 1.268pg/mL (AUC 0.797, 95% CI 0.704-0.890, sensitivity 75.0%, specificity 72.7%, **Figure 4A** and 4G). The optimum diagnostic cutoff for IP-10 was 9.851pg/mL (AUC 0.665, 95% CI 0.551-0.778, sensitivity 52.3%, specificity 75.0%, **Figures 4B, G**). The optimum diagnostic cutoff for PDGF-BB was 1229pg/mL (AUC 0.669, 95% CI 0.555-0.784, sensitivity 65.9%, specificity 63.6%, **Figures 4C, G**). Totally, IL-6 demonstrated exceptional diagnostic performance in childhood obesity.

All values rose when the two or three tests were combined (**Figure 4G**). ROC analysis showed that testing of both IL-6 and IP-10 increased the diagnostic accuracy for childhood obesity (AUC 0.816, 95% CI 0.727-0.905, sensitivity 72.7%, specificity 75.0%, **Figures 4D, G**). Diagnostic accuracy of the combination of IL-6 and PDGF-BB increased than test alone (AUC 0.810, 95% CI 0.719-0.902, sensitivity 72.7%, specificity 77.3%, **Figures 4E, G**). The combination of IL-6, IP-10 and PDGF-BB shown the greatest diagnostic accuracy in childhood obesity (AUC 0.830, 95% CI 0.744-0.917, sensitivity 70.5%, specificity 75.0%, **Figures 4F, G**). We also calculate the positive predictive value (PPV), negative predictive value (NPV), positive likelihood ratio (PLR), negative likelihood ratio (NLR) of individual test and their combination (**Figure 4G**). However, whether these indicators can be used as early predictors of childhood obesity still requires further research.

## 4 Discussion

Obesity is associated with chronic low-grade inflammation. An imbalance in the levels of inflammatory factors may contribute to obesity by fostering chronic inflammation within adipose tissue. Cytokines are a group of small molecular proteins or peptides that demonstrate biological activity. They can be classified into pro-inflammatory and anti-inflammatory types (15, 16). Numerous cytokines exert regulatory effects on the progression of obesity, either by promoting or inhibiting its development (17).

In this study, we conducted a systematic observation of changes in 48 cytokines in childhood obesity. The findings revealed that, in comparison to normal weight children, several cytokines, including MIP-1b, PDGF-BB, IP-10, IL-6, IL-9, and TNF-β, exhibited elevated levels in the serum of children with obesity. Interestingly, TNF-β instead of TNF-α showed a tendency higher in patients with childhood obesity than control group. Although TNF-α is a well-known pro-inflammatory cytokine associated with obesity, TNF-β also plays a significant role in inflammatory processes. Both cytokines are involved in the pathogenesis of obesity-related inflammation, but TNF-β may have distinct roles in modulating immune responses and inflammation in adipose tissue. Further studies are needed to elucidate the specific roles of TNF-β in childhood obesity. Notably, IL-6 demonstrated the most significant elevation. IL-6, a multifunctional cytokine, can be secreted by various cell types, including immune cells, fibroblasts, endothelial cells, and adipocytes, exhibiting both pro-inflammatory and anti-inflammatory properties, playing a role in regulating body weight and lipid metabolism (18). In the context of obesity, IL-6 is intricately associated with chronic inflammatory processes and contributes to the inhibition of hepatic insulin signaling through the induction of SOCS3 protein expression. This involves direct impediment of insulin receptor and insulin receptor substrates-1,2 activation and degradation, which are pivotal in the pathogenesis of insulin resistance (19). The primary source of elevated circulating IL-6 levels in obesity emanates from immune cells within adipose tissue, with heightened IL-6 release being closely linked to obesity (20). In obesity mouse model, administration of the IL-6 receptor (IL-6R) inhibitor tocilizumab (TCZ) has been shown to significantly mitigate weight gain, regulate adipose tissue hypertrophy, enhance insulin sensitivity, and promote improved glucose tolerance (21). Repression of IL-6R and IL-6 could be considered as a promising therapeutic approach for the management of obesity (22).

Meanwhile, there are also reports in the literature suggesting that IL-6 may have divergent effects (23). A mounting body of evidence indicates that IL-6 serves as a pivotal regulator of energy and glucose homeostasis. It has been demonstrated that IL-6 modulates energy and glucose homeostasis by activating the transcription factor STAT3 through a shared molecular pathway with leptin (24). Moreover, IL-6 can effectively suppress appetite and improve peripheral glucose homeostasis by activating STAT3 signaling in hypothalamic neurons (25). Therefore, the upregulation of IL-6 during obesity may represent an adaptive mechanism aimed at enhancing insulin production and ameliorating glucose tolerance to counteract obesity-related insulin resistance. While ROC analysis demonstrates the diagnostic potential of IL-6 and other cytokines, it is critical to acknowledge that BMI remains the gold standard for obesity diagnosis due to its simplicity and clinical feasibility. The clinical utility of these cytokines lies not in replacing BMI but in providing mechanistic insights into obesity-related inflammation. For instance, IL-6’s dual role in promoting insulin resistance and regulating energy homeostasis highlights its potential as a therapeutic target. Future studies should explore whether cytokine profiles can identify high-risk individuals before BMI elevation occurs, enabling earlier intervention.

The lack of direct correlation between cytokine levels and HDL/LDL in this study does not negate the potential relationship between chronic inflammation and lipid metabolism. It is possible that the interplay between cytokines and lipid profiles is complex and influenced by multiple factors. Future studies with larger sample sizes and more comprehensive lipid profiling may provide deeper insights into this relationship.

Another interesting aspect to consider is the role of CD40 in obesity-related inflammation. Because the Bio-Plex Pro Human Cytokine Screening 48-plex Panel kit we used does not contain CD40, there is no way for our results to reflect the important role of CD40 in childhood obesity, but there are previous reports in the literature on the importance of CD40 (26). CD40, a transmembrane protein expressed on various cells including adipocytes and immune cells, has been reported to play a significant role in the production of inflammatory cytokines. Poggi and Lutgens demonstrated that CD40 engagement leads to high levels of inflammatory cytokines, which may contribute to the chronic low-grade inflammation observed in obesity. Further research is needed to explore the potential role of CD40 in childhood obesity and its interaction with other inflammatory pathways.

At present, childhood obesity has received extensive social attention. Some management strategies for childhood obesity have also been developed clinically, dedicated to providing comprehensive care for patients with obesity, including nutritional therapy, physical activity, behavioral modification and medical interventions (27). However, once obesity occurs, management is relatively difficult. Intervention in the early stage when obesity is likely to occur but has not yet occurred is more significant than treatment. We detect cytokine levels in patient serum to provide a theoretical basis for exploring early markers of childhood obesity and relevant strategies for the daily clinical work of preventing childhood obesity.

## 5 Study limitations

Limitations of this study include the relatively small sample size, which may limit the generalizability of the findings. Additionally, the cross-sectional design does not allow for causal inferences regarding the relationship between cytokine levels and obesity. Longitudinal studies with larger cohorts are needed to validate these results and explore the temporal dynamics of cytokine changes in obesity. Furthermore, the study focused on a specific panel of cytokines, and other potentially relevant molecules were not assessed. Future research should aim to include a broader range of inflammatory markers and investigate their interactions in the context of obesity.

## Data Availability

The original contributions presented in the study are included in the article/**Supplementary Material**. Further inquiries can be directed to the corresponding author.

## References

[1] Afshin A Forouzanfar MH Reitsma MB Sur P Estep K Lee A. Health effects of overweight and obesity in 195 countries over 25 years. New Engl J medicine. (2017) 377:13–27. doi: 10.1056/NEJMoa1614362 PMC5477817 28604169

[2] Vick LV Collins CP Khuat LT Wang Z Dunai C Aguilar EG. Aging augments obesity-induced thymic involution and peripheral T cell exhaustion altering the “obesity paradox. Front Immunol. (2022) 13:1012016. doi: 10.3389/fimmu.2022.1012016 36776393 PMC9910174

[3] Hill L Schwickert TA. NR4As apply brakes on the B cell response. Nat Immunol. (2020) 21:1137–9. doi: 10.1038/s41590-020-0775-5 32868931

[4] The Lancet Diabetes E. Childhood obesity: a growing pandemic. Lancet Diabetes Endocrinol. (2022) 10:1. doi: 10.1016/S2213-8587(21)00314-4 34863372 PMC9765420

[5] Dai W Liu X Su H Li X Xu Y Yu Y. Influence of adipose tissue immune dysfunction on childhood obesity. Cytokine Growth Factor Rev. (2022) 65:27–38. doi: 10.1016/j.cytogfr.2022.04.008 35595599

[6] Hotamisligil GS. Inflammation and endoplasmic reticulum stress in obesity and diabetes. Int J Obes (Lond). (2008) 32 Suppl 7:S52–4. doi: 10.1038/ijo.2008.238 PMC2885768 19136991

[7] Jiao P Chen Q Shah S Du J Tao B Tzameli I. Obesity-related upregulation of monocyte chemotactic factors in adipocytes: involvement of nuclear factor-kappaB and c-Jun NH2-terminal kinase pathways. Diabetes. (2009) 58:104–15. doi: 10.2337/db07-1344 PMC2606857 18835938

[8] Wu H Ballantyne CM. Metabolic inflammation and insulin resistance in obesity. Circ Res. (2020) 126:1549–64. doi: 10.1161/CIRCRESAHA.119.315896 PMC7250139 32437299

[9] Lin TY Chiu CJ Kuan CH Chen FH Shen YC Wu CH. IL-29 promoted obesity-induced inflammation and insulin resistance. Cell Mol Immunol. (2020) 17:369–79. doi: 10.1038/s41423-019-0262-9 PMC7109060 31363171

[10] Weisberg SP McCann D Desai M Rosenbaum M Leibel RL Ferrante AW Jr. Obesity is associated with macrophage accumulation in adipose tissue. J Clin investigation. (2003) 112:1796–808. doi: 10.1172/JCI200319246 PMC296995 14679176

[11] Hotamisligil GS Shargill NS Spiegelman BM. Adipose expression of tumor necrosis factor-alpha: direct role in obesity-linked insulin resistance. Sci (New York NY). (1993) 259:87–91. doi: 10.1126/science.7678183 7678183

[12] Song MY Kim SH Ryoo GH Kim MK Cha HN Park SY. Adipose sirtuin 6 drives macrophage polarization toward M2 through IL-4 production and maintains systemic insulin sensitivity in mice and humans. Exp Mol medicine. (2019) 51:1–10. doi: 10.1038/s12276-019-0256-9 PMC6529411 31113929

[13] Arslan N Erdur B Aydin A. Hormones and cytokines in childhood obesity. Indian pediatrics. (2010) 47:829–39. doi: 10.1007/s13312-010-0142-y 21048235

[14] Larque E Labayen I Flodmark CE Lissau I Czernin S Moreno LA. From conception to infancy - early risk factors for childhood obesity. Nat Rev Endocrinol. (2019) 15:456–78. doi: 1038/s41574-019-0219-1 10.1038/s41574-019-0219-1 31270440

[15] Ullah A Singla RK Batool Z Cao D Shen B. Pro- and anti-inflammatory cytokines are the game-changers in childhood obesity-associated metabolic disorders (diabetes and non-alcoholic fatty liver diseases). Rev Endocr Metab Disord. (2024) 25:783–803. doi: 10.1007/s11154-024-09884-y 38709387

[16] Li W Chen F Gao H Xu Z Zhou Y Wang S. Cytokine concentration in peripheral blood of patients with colorectal cancer. Front Immunol. (2023) 14:1175513. doi: 10.3389/fimmu.2023.1175513 37063892 PMC10098211

[17] Li Y Wang X Zhang Z Shi L Cheng L Zhang X. Effect of the gut microbiome, plasma metabolome, peripheral cells, and inflammatory cytokines on obesity: a bidirectional two-sample Mendelian randomization study and mediation analysis. Front Immunol. (2024) 15:1348347. doi: 10.3389/fimmu.2024.1348347 38558794 PMC10981273

[18] Koc G Doran T Uygur MM Kirac D. Obesity is associated with IL-6 gene polymorphisms rs1800795 and rs1800796 but not SOCS3 rs4969170. Mol Biol Rep. (2023) 50:2041–8. doi: 10.1007/s11033-022-08129-y 36538174

[19] Kim YD Kim YH Cho YM Kim DK Ahn SW Lee JM. Metformin ameliorates IL-6-induced hepatic insulin resistance via induction of orphan nuclear receptor small heterodimer partner (SHP) in mouse models. Diabetologia. (2012) 55:1482–94. doi: 10.1007/s00125-012-2494-4 22349108

[20] Illan-Gomez F Gonzalvez-Ortega M Orea-Soler I Alcaraz-Tafalla MS Aragon-Alonso A Pascual-Diaz M. Obesity and inflammation: change in adiponectin, C-reactive protein, tumour necrosis factor-alpha and interleukin-6 after bariatric surgery. Obes Surg. (2012) 22:950–5. doi: 10.1007/s11695-012-0643-y 22527592

[21] Mondanelli G Albini E Orecchini E Pallotta MT Belladonna ML Ricci G. Pathogenetic interplay between IL-6 and tryptophan metabolism in an experimental model of obesity. Front Immunol. (2021) 12:713989. doi: 10.3389/fimmu.2021.713989 34394118 PMC8361489

[22] Ji Z Wu S Xu Y Qi J Su X Shen L. Obesity promotes EAE through IL-6 and CCL-2-mediated T cells infiltration. Front Immunol. (2019) 10:1881. doi: 10.3389/fimmu.2019.01881 31507583 PMC6718738

[23] Wueest S Konrad D. The controversial role of IL-6 in adipose tissue on obesity-induced dysregulation of glucose metabolism. Am J Physiol Endocrinol Metab. (2020) 319:E607–e13. doi: 10.1152/ajpendo.00306.2020 32715746

[24] Mauer J Denson JL Bruning JC. Versatile functions for IL-6 in metabolism and cancer. Trends Immunol. (2015) 36:92–101. doi: 10.1016/j.it.2014.12.008 25616716

[25] Timper K Denson JL Steculorum SM Heilinger C Engstrom-Ruud L Wunderlich CM. IL-6 Improves Energy and Glucose Homeostasis in Obesity via Enhanced Central IL-6 trans-Signaling. Cell Rep. (2017) 19:267–80. doi: 10.1016/j.celrep.2017.03.043 28402851

[26] Lutgens E Poggi M Weber C. CD40L-CD40 fuel ignites obesity. Thromb Haemost. (2010) 103:694–5. doi: 10.1160/TH10-03-0146 20216980

[27] Cuda S Censani M O’Hara V Paisley J Kharofa R Conroy R. Special considerations for the child with obesity: An Obesity Medicine Association (OMA) clinical practice statement (CPS) 2024. Obes Pillars. (2024) 11:100113. doi: 10.1016/j.obpill.2024.100113 38953014 PMC11216014

