# Supplementary material for: Cytokine concentration in peripheral blood of patients with childhood obesity
Source: Front Immunol. 2025 May 30;16:1606375. doi: 10.3389/fimmu.2025.1606375 (PMC12162999; doi:10.3389/fimmu.2025.1606375)
Supplement: Supplementary file 1 [file DataSheet1.pdf]

Cytokine concentration in peripheral blood of patients with childhood obesity

Jia Mai[1], Min Wang[1], Ying Guo[1], Ling Yang[1], HongJian Xie[1], Lan Mei[1], ZiYao Zhu[1], XiaoJuan Liu [1,*]

1 Department of Laboratory Medicine, West China Second University Hospital, Sichuan University; key laboratory of birth defects and related diseases of women and children (Sichuan University), Ministry of Education, Chengdu, Sichuan, China;

Correspondence: Department of Laboratory Medicine, West China Second University Hospital, Sichuan University, No. 20, Section 3, Ren Min Nan Lu, 610041, Chengdu, Sichuan, China;

Email: maggielxj1979@163.com (XiaoJuan Liu)

Supplementary Table S1: Representative performance characteristics

	Assay working range, pg/ml		Assay sensitivity, pg/ml	Mean Intra-Assay %CV	Mean Inter-Assay %CV	Singleplex Bead Region
**Analyte**	LLOQ	ULOQ	LOD			
**Basic FGF**	3.26	3,341	2.54	3.1	2.4	44
**CTACK**	2.10	15,656	0.82	2.7	5.2	72
**Eotaxin**	0.14	2,281	0.05	4.4	1.2	43
**G-CSF**	6.35	104,106	3.63	3.1	4.0	57
**GM-CSF**	0.48	7,846	0.19	4.3	2.2	34
**GRO-α**	21.05	31,255	13.45	2.6	7.9	61
**HGF**	8.76	143,513	7.09	2.6	2.9	62
**IFN-α2**	0.95	15,569	0.46	3.3	4.4	20
**IFN-γ**	1.57	25,665	1.05	3.1	3.6	21
**IL-1α**	3.73	61,154	6.65	3.5	4.9	63
**IL-1β**	0.29	4,672	0.24	3.6	3.2	39
**IL-1ra**	6.21	34,949	3.16	4.7	5.1	25
**IL-2**	1.29	21,178	0.75	1.7	2.5	38
**IL-2Rα**	1.48	24,270	1.65	3.4	4.8	13
**IL-3**	0.13	2,139	0.13	5.0	3.9	64
**IL-4**	0.19	3,064	0.09	3.2	1.9	52
**IL-5**	3.63	59,499	0.86	2.3	2.3	33
**IL-6**	0.38	6,244	0.34	2.2	3.0	19
**IL-7**	1.92	31,475	1.22	2.7	3.9	74
**IL-8**	0.85	13,992	0.36	3.2	2.8	54
**IL-9**	3.62	31,527	1.08	2.6	7.1	77
**IL-10**	1.06	17,427	0.69	2.3	3.4	56
**IL-12 (p40)**	14.68	240,582	6.39	4.5	2.4	28

**IL-12 (p70)**	1.43	23,425	0.78	3.3	2.9	75
**IL-13**	0.31	5,157	0.22	3.1	2.7	51
**IL-15**	12.42	203,426	12.82	2.8	4.1	73
**IL-16**	1.20	19,639	0.25	2.5	3.0	27
**IL-17A**	2.44	39,972	1.16	2.4	1.4	76
**IL-18**	0.66	10,892	0.31	2.9	2.2	42
**IP-10**	3.41	34,953	1.43	2.8	6.0	48
**LIF**	3.86	53,806	2.05	2.5	4.7	29
**MCP-1 (MCAF)**	0.53	8,755	0.44	3.2	3.4	53
**MCP-3**	0.48	4,899	0.24	4.4	4.2	26
**M-CSF**	0.75	12,290	0.27	2.4	3.6	67
**MIF**	2.70	44,168	2.45	3.4	4.7	35
**MIG**	3.16	32,365	1.39	4.4	4.2	14
**MIP-1α**	0.12	1,218	0.06	4.5	4.2	55
**MIP-1β**	1.41	1,439	1.41	3.4	2.5	18
**B-NGF**	0.47	7,655	0.23	2.9	3.9	46
**PDGF-BB**	7.12	37,133	2.96	3.3	9.7	47
**RANTES**	16.72	24,467	3.98	3.0	6.7	37
**SCF**	1.82	29,899	0.99	4.1	2.6	65
**SCGF-β**	82.11	1,345,200	141.77	2.3	3.8	78
**SDF-1α**	7.54	9,381	2.44	2.2	5.4	22
**TNF-α**	3.33	54,566	1.13	3.5	3.0	36
**TNF-β**	0.8	13,186	0.38	3.0	4.7	30
**TRAIL**	1.78	29,188	0.89	3.2	4.5	66
**VEGF-A**	18.01	149,830	10.16	2.8	8.5	45

The LLOQ,ULOQ, LOD, and inter-assay precision %CV are mean data determined from three independent multiplex assays in a serum-based matrix. Intra-assay %CV is derived from one representative assay. LLOQ and ULOQ are defined as the boundary standard curve points

within which the performance specifications of individual standard points were met for a 10% intra-assay CV and recovery range of 70-130%. Data were generated using the magnetic workflow with the Bio-Plex Pro Wash Station.
